# Electroacupuncture at ST25 mediated glial cells pruning of pancreatic TRPV1 neural synapse responds to neuropathy-associated beta cell dysfunction

**DOI:** 10.1186/s13020-025-01099-w

**Published:** 2025-05-16

**Authors:** Yun Liu, Jiahui Xie, Zhi Yu, Meirong Gong, Qian Li, Guanhu Yang, Bin Xu, Tiancheng Xu

**Affiliations:** 1 Key Laboratory of Acupuncture and Medicine Research of Ministry of Education, Nanjing University of Chinese Medicine, Nanjing, China; 2 Research Department, Swiss University of Traditional Chinese Medicine, Bad Zurzach, Switzerland; 3 Department of Specialty Medicine, Ohio University, Athens, 43055 USA

**Keywords:** β cell, Glial cells, TRPV1 nerve axon, CGRP, Electroacupuncture

## Abstract

Electroacupuncture (EA) is verified to modulate glycemic changes in T2DM, and this is partially related to sensory neurotransmitters. However, EA-mediated communication mechanism between them and acupoint specificity have not been fully clarified. Thus, we replicated the diabetic rat model induced by a high-fat diet/streptozotocin (HFD-STZ), and investigated the alleviating effects on insulin resistance (IR) and inflammation severity after EA at ST25. We also compared the effect difference of EA at ST37. Furthermore, we studied the changes of pancreatic sensory neurotransmitters and β cells (and their surrounding components) in detail. Serum glucose, insulin, IR, TNF-α and IL-10 were significantly elevated in model rats, and β cell function was impaired, which reversed by EA at ST25 or ST37 to varying degrees. EA at ST25 can enhance the expression of calcitonin gene-related peptide (CGRP), attenuate transient receptor potential vanilloid 1(TRPV1) and correct the secretion mismatch between them, while EA at ST37 has no such effect. Subsequently, EA at ST25-mediated TRPV1-CGRP-β cell circuit demonstrates an advantage in regulating glucose metabolism via direct insulin inhibition by CGRP. EA at ST25 rather than ST37 regulates the activity of peri-ilset glial cells and macrophages, playing a neuro-protective role and controlling inflammation. EA at ST37 exhibits its partial therapeutic effect on T2DM as it improves serum GLP-1. It also implies that the dominant target organ of ST37 may not be the pancreas, but other associated viscera. Hence, our study elucidates the EA-mediated glial cell via TRPV1-CGRP pathway regulation of β cell dysfunction after nerve lesion, and the hypoglycemic effect of ST25 is significantly better than that of ST37.

**Graphical Abstract:**

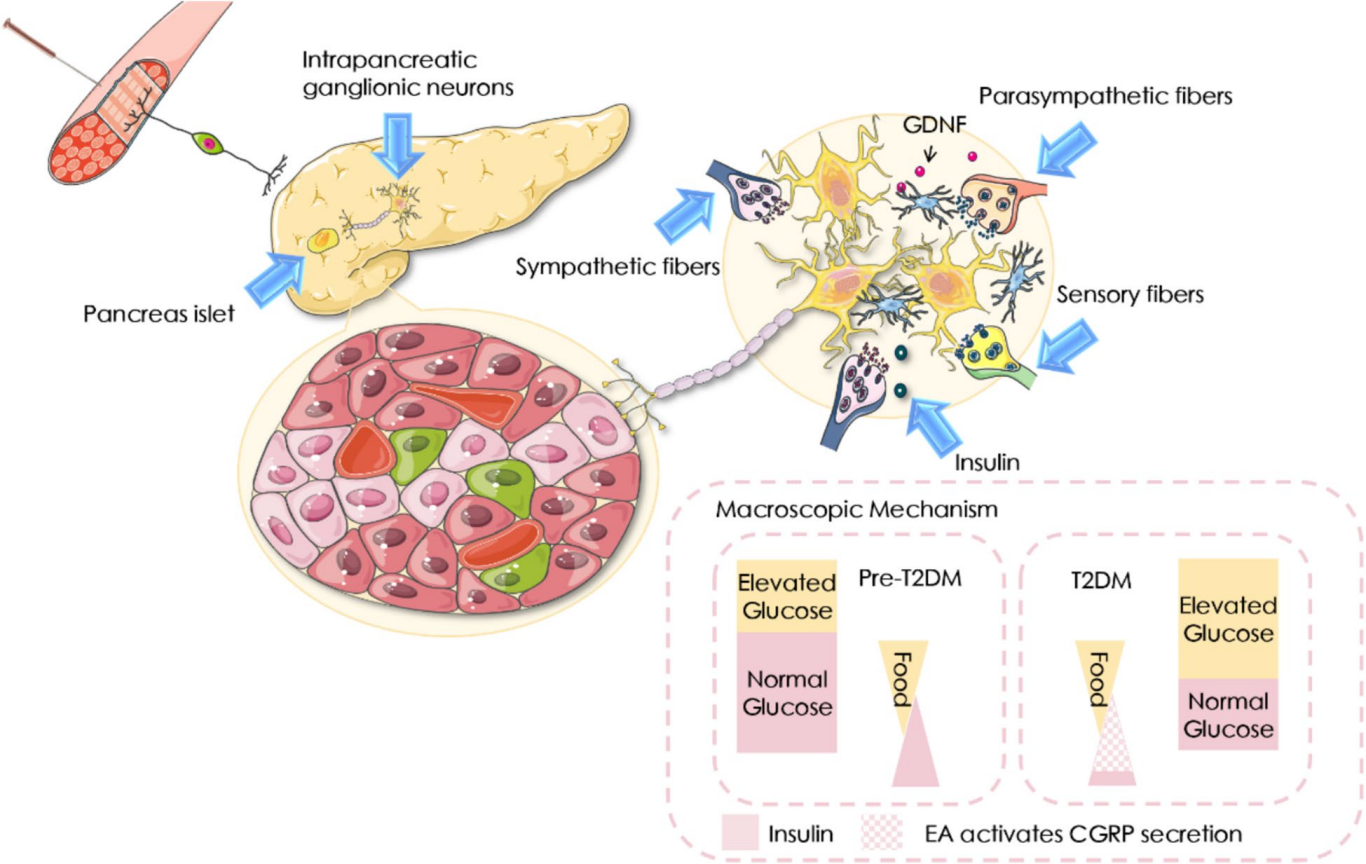

**Supplementary Information:**

The online version contains supplementary material available at 10.1186/s13020-025-01099-w.

## Introduction

T2DM is caused by the insufficient response of islet β cells and adipose tissue to excess chronic nutrients, leading to insulin resistance and metabolic stress [1]. Effective communication between β cells and other tissues is the basis of a dynamic balance of glucolipid metabolism and energy stability. Gray [2] pointed out that during the onset of T2DM, the pancreatic secretion of calcitonin gene-related peptide (CGRP) decreased and cannot suppress the pathological insulin secretion. Therefore, a new paradigm with CGRP as the monitoring core becomes a new prospect for the diagnosis and treatment of T2DM. The extensive plasticity of CGRP and its associated positive neurons provide a possibility for the treatment of pathological conditions [3]. Especially, CGRP cascades released after activation of transient receptor potential vanilloid type 1(TRPV1) positive neurons can directly inhibit insulin production. Moreover, in the periphery, TRPV1 can control energy intake and maintain consumption balance by controlling hormone secretion [4]. Tang [5] pointed out that both TRPV1 and CGRP significantly augmented in diabetes to inhibit the over secretion of insulin. However, different perspectives still exist. Razavi [6] detected that the TRPV1 receptor affects the local feedback between sensory neurons and β cells, Gram [7] suggested that there was a physiological limit to the TRPV1 receptor. Nevertheless, these results suggested that TRPV1 dysfunction may trigger or aggravate the process of T2DM. Therefore, we will focus on the changes of TRPV1 in the pancreas as the basis for the role of TRPV1 in diseases related to glucose and lipid metabolism.

The sensitivity of β cells to physiological glucose concentration mainly relies on the regulation of insulin secretion by pancreatic autonomic nerve. Hyperglycemia leads to pancreatic nerve remodeling. This dynamic change of peripheral innervation may be a new target for the treatment of diabetes[8]. However, the innervation of the autonomic nerve branch in the pancreas and its related effects are unknown. The β cells show varying degrees of damage due to the complex endocrine unit of pancreatic islet and the heterogeneity of β cells[9]. Thus, two cognitions have been developed to regulate the function of β cells. One theory emphasizes direct stimulation of β cells by autonomic nerves or to stimulate β cells directly via autonomic nerves[10]. Another is to inhibit or disinhibit the effects of other components such as glial cells and intestinal cells on β cells [11]. In this study, we will focus on the latter part. Establishing precise synaptic connections is essential for the proper functioning of the nervous system. Neurons can selectively strengthen active synapses while phasing out those that are inactive [12]. This process is critical for maintaining neural circuit integrity and plasticity. The elimination of inactive synapses occurs in the context of competition with other, more active connections. Synapses, as critical junctures for the exchange and transmission of neuronal information, play an essential role in the connectivity and functionality of neural loops [13]. The processes of synapse formation, elimination, and remodeling are integral to this dynamic. Microglia participate in the formation and maturation of neuronal loop connections [14]. Microglia are multifunctional, contributing to the regulation of neurogenesis [15], the stimulation of synaptogenesis [16], the pruning of immature synapses [17], the promotion of neuronal apoptosis [18], and the phagocytosis of cellular debris [19]. Glial cells are also indispensable for phagocytosis, clearance, and immunosurveillance within the central nervous system. The pre-synaptic janus kinase 2 (JAK2) detects "punishment" signals from active synapses, triggering the clearance of inactive synapses [20]. While this glial cell-mediated neuroprotection is recognized, the extent to which the synaptic pruning signaling pathway governs pancreatic glial cell phagocytosis in the construction of pancreatic neural synapses remains to be further explored. Our previous work has demonstrated a dynamic link between autonomic nerve stimulation and insulin secretion during hyperglycemia, and preliminarily indicated that EA at ST25 can partially repair the neurophysiological damage under hyperglycemia[21]. Thus, we further validated the effects of different neuronal subtypes on pancreatic reinnervation, β cells function and neuro-immune response. Meanwhile, leveraging the clinical evidence that EA at ST37 can improve lipid metabolism[22], this study also comparatively investigated the differences in the effects of EA at various acupoints on pancreatic metabolism and the underlying mechanisms.

## Materials and methods

### Animal model organization

**Fig. 1:**
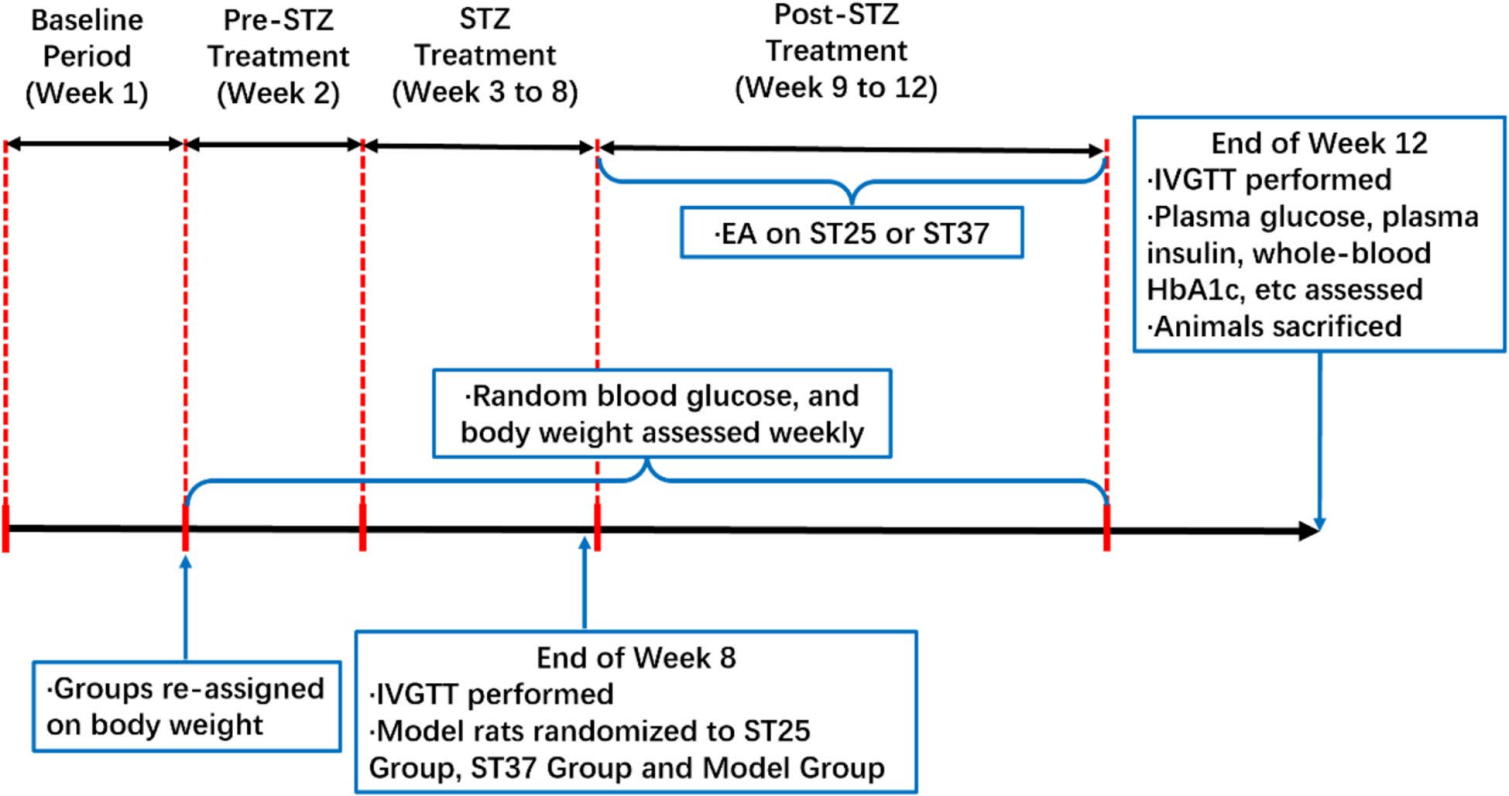
Schedule of the experimental procedures. EA, electroacupuncture; ST25, acupoint Tianshu; ST37, acupoint Shangjuxu; STZ, streptozotocin; IVGTT, intravenous glucose tolerance test; HbA1c, glycated hemoglobin

A high-fat diet integrated with streptozotocin (low dose, multiple doses) is a mature preparation method for a diabetic rat model that had been tested to replicate the metabolic dysfunctions associated with human T2DM [23]. Sprague–Dawley (SD) rats routine feeding at the age of 7 weeks were all purchased from the Nanjing University of Chinese Medicine (No. 1100112011052760, under grant SCXK (JING)2016–0006). Their living environment was maintained on a periodic cycle principle (alternating light/dark conditions every 12 ± 1 h; controlled temperature maintained at 20 °C to 24 °C; and relative humidity 55%-65%). Rats were given sufficient food and water regularly, divided into cages and fed separately. Then, five rats were randomly parted into a group (normal control group, model group, ST25 group and ST37 group) with random number marking. High fat diet (HFD) fed rats were injected with streptozotocin (STZ) [24] to achieve diet-chemically induced type 2 diabetes mellitus (T2DM). STZ was added to the citric buffer (0.1 mol/L, pH 4.2) which was freshly prepared and injected. All rats except the normal control group were injected of STZ (35 mg/kg, i.p., Sigma, St. Louis, MO, USA). The end time of intervention and eat were recorded routinely. After 48 h, the vein blood collection was performed. Random blood glucose levels were measured by a glucometer (Roche Diagnostics, Mannheim, Germany). If the blood glucose level was over 16.7 mmol/L and maintained for more than two weeks, the model was successful [16, 25]. Keeping track of weekly weight changes and random blood glucose levels. During the whole intervention period, only the normal control group was given the standard diet, the other three groups were always given HFD mixed with carbohydrate, protein, and fat, which the calories combination proportion was 17%, 25%, and 58%, respectively, and strictly controlled the proportion of calories intake of the rats to induce IR production [26]. The rats in the EA groups and the model group who failed to construct the model were killed by cervical dislocation. The manipulation was performed according to Fig. 1. Experimental procedure follows the Principles of Laboratory Animal Care and the Guide for the Care and Use of Laboratory Animals published by the National Science Council, China (under grant 202006A016).

### Blood test and tissue analysis

The experiment was finished at the 12 week, with blood and tissue sampling collected. The centrifuged serum was temporarily preserved at − 80 °C for later use. After the pancreas was removed, they were quickly rinsed and placed in 10% paraformaldehyde or − 80 °C for later use. The metabolic parameters, such as fasting serum insulin, glycated hemoglobin, leptin, and GLP-1, were measured by rat ELISA kit. The detection methods of tumor necrosis factor α (TNF-a), interleukin 10 (IL-10), and IL-1β were the same as above.

### Antibodies

After the membrane is sealed, the corresponding sites are bound by the primary antibody and the secondary antibody respectively. The specific information of the primary antibody is shown in Table S1. The secondary antibodies used were anti-rabbit IgG, HRP-linked antibody (1:2000, Cell Signaling Technology), and Anti-mouse IgG, HRP-linked antibody (1:2000, Cell Signaling Technology).

### Heart rate variability (HRV) collection and analysis

The rats were under anesthesia invariably during the whole procedure (20% Ulatan 0.5 ml/100 g, i.p.) [27]. The connection method of the rat electrocardiogram (ECG) recording electrode is as follows: an acupuncture needle is pierced under the skin and fixed on the needle with a clip. The left lower limb is connected to the positive pole and the right upper limb is connected to the negative pole [28]. After the experiment, the rats were immediately given overdose of anesthetic and sacrificed.

HRV parameters were sampled by PowerLab (Micro1401-3, CED, UK) and the frequency of sample was 2 K/s. HRV module of Labchart 8.0 software (AD Instruments, Colorado Springs, CO, USA) was used to analyze power in high-frequency (HF norm) and low-frequency (LF norm) in normalized units, and LF/HF. HRV index changes during before, during and after acupuncture were compared. The frequency domain index reflecting vagal nerve activity in HRV is high frequency (HF), while low frequency (LF) is considered to reflect sympathetic nerve activity to a greater extent. HRV LF power and HF power are expressed in normalized units(nu). The LF/HF ratio reflects the balance of parasympathetic activity.

### Hematoxylin and eosin staining

Hematoxylin and eosin (HE) staining refers to histological procedures. For hematoxylin and eosin (HE) staining, refer to histological procedures. After blunt dissection of the pancreas, 4% paraformaldehyde was treated and fixed with paraffin embedding. After blunt dissection of the pancreas, 4% paraformaldehyde was treated and fixed with paraffin embedding. Place the slices on a rotary slicer (Leica, Germany), the thickness is 8 μm, and stick them on the slides. HE staining under a light microscope (Olympus, Japan) was used to evaluate the pathological changes of pancreas.

### Western blot analysis

After full abdominal anesthesia, the pancreas was quickly dissected and about 400 mg of tissue was removed. A 1 mL lysis buffer was prepared in advance: a combination of protease inhibitors and RIPA buffer (Thermo Scientific Inc, Waltham, USA). Part of the tissue sample (200 mg of tissue) was placed in it and centrifuged for 0.5 h, and the parameter was set to 14,000 rpm. Ultimately, the protein concentration was determined by the BCA Protein Assay Kit (Thermo Scientific Inc, Waltham, USA). Protein samples of 20 μg were successively extracted and then added to sodium dodecyl sulfate–polyacrylamide gel (12% separation gel and 5% concentration gel) for electrophoresis. The voltage parameters were changed according to the electrophoresis condition, which was 70 V for 20 min, 90 V for 60 min and 110 V for 20 min. Trans-Blot Turbo Transfer System (Bio-Rad, Hercules, USA) was used as a carrier to transfer the protein bands to the polyvinylidene fluoride (PVDF) membrane. Bovine Serum Albumin (BSA, 5%) was used for blocking, and the duration was controlled for 1 h. Repeat the above operation for each layer for about 1 h. Then transfer to a Tris-buffered saline containing Tween (TBST) cleaning membrane. Refer to Table S1 to configure the primary antibody concentration. Drop antibody into the corresponding band and incubate at 4 °C for 16 h. Then transferred it to TBST for rewashing, and then the secondary antibody was added at 28 °C for 60 min. Calculated by enhanced chemiluminescence detection. Image J (NIH, Bethesda, USA) was used to quantify the gray values of immunoreactive protein bands.

### Immunofluorescence staining

The corresponding frozen sections were prepared for subsequent staining steps. Pancreatic tissues soaked overnight in 4% paraformaldehyde were transferred to 30% sucrose containing 0.1 M PBS (BioSharp Life Sciences, Beijing, China) and dehydrated until the tissue completely sank. The ambient temperature should be maintained at 4 °C. Adjust to the appropriate temperature and slice into sections, about 10 μm in thickness, and stick them on the slide. 0.2% Triton X-100 (Sigma-Aldrich Shanghai Trading Co., Ltd. Shanghai, China) was used to break the film and treat the sections for 10 min. The Sea Block buffer (Thermo Scientific Inc, Waltham, USA) was then used to permeate for 1 h. The primary anti-insulin antibody (1:100, Signalway Antibody, Nanjing, China) was dropped and transferred to 4 °C for full response overnight, followed by incubation with secondary antibodies as Alexa Fluor 488 (goat anti-rabbit, 1:500, Abcam, Cambridge, UK), and Alexa Fluor 594 (goat anti-mouse, 1:500, Abcam, Cambridge, UK), for 1 h at 37 °C. The appropriate secondary antibody is combined to obtain the desired image (Olympus BX60 Darkfield DIC metallographic microscope, Tokyo, Japan). Ultimately, drop 0.1 M PBS to fully clean the residual liquid on the tissues, and cover the cover glass. Antifade Mounting Medium with DAPI (Solarbio S2110, Beijing, China) was added before observation.

### EA stimulation

EA is a pulsating electric current stimulation applied to specific acupoints through acupuncture needles [29]. After isoflurane (2% ~ 5%) was fully anesthetized, EA was performed at ST25 (Tianshu, located 5 mm lateral to the intersection between the upper 2/3rd and the lower 1/3rd in the line joining the xiphoid process and the upper border of the pubic symphysis) or ST37 (Shangjuxu, located 5 mm below the knee joint and 1 mm lateral to the margo anterior tibiae). EA was given to rats in the treatment groups, while the model group did not receive it. Two stainless steel acupuncture needles (Hwato, 20162270970, Suzhou, China) with a diameter of 0.2 mm were selected and combined. The needles were inserted vertically with depth controlled at 5 mm. Using Hans-100A (Han Acuten, WQ1002F, Beijing, China) instrument to control the given current, the parameter was stabilized at 2 mA, 2/15 Hz. 0.5 h. Stimulation was given every day, 6 times a week, as a course of treatment. It is operated by skilled and licensed acupuncturists throughout the four weeks. Based on our team's previous research, four weeks of electroacupuncture can effectively improve fat redistribution[30] and reduce the level of pancreatic inflammation and metabolic abnormalities in rats[31]. Additionally, when HRV was recorded, acupuncture intervention was performed for 2 min after baseline stabilization was observed.

### Paraventricular nucleus (PVN) targeted injection

In accordance with the guidelines from George Paxinos and Charles Watson's stereotactic atlas of the rat brain, the flat skull fixation method was employed [32]. The rats were secured using three reference points: the bilateral inner ear orifices and the incisors. The skull was fully exposed, and the superficial connective tissue was carefully removed using an absorbent cotton ball. The position of the bregma was subsequently identified, and the corresponding coordinates on the x, y, and z axes were recorded as the reference zero point. The PVN of the hypothalamus was marked with a marker pen, located 1.8 mm posterior to the bregma along the midline, with bilateral offsets of 0.4 mm and a depth of 7.9 mm.

### Data analysis

The experimental data were presented as mean ± standard error values. Paired-sample Student’s t-test to compare the values before and after electroacupuncture intervention, and one-way ANOVA was used for comparison of multiple groups. The above data were calculated using SPSS 22.0 software (IBM, Armonk, New York, USA) and GraphPad Prism 8.0 (GraphPad Inc., La Holla, CA, USA). P < 0.05 indicates a significant difference.

## Results

### EA at ST25 or ST37 improved pancreatic glucolipid metabolismand controlled inflammatory status

**Fig. 2:**
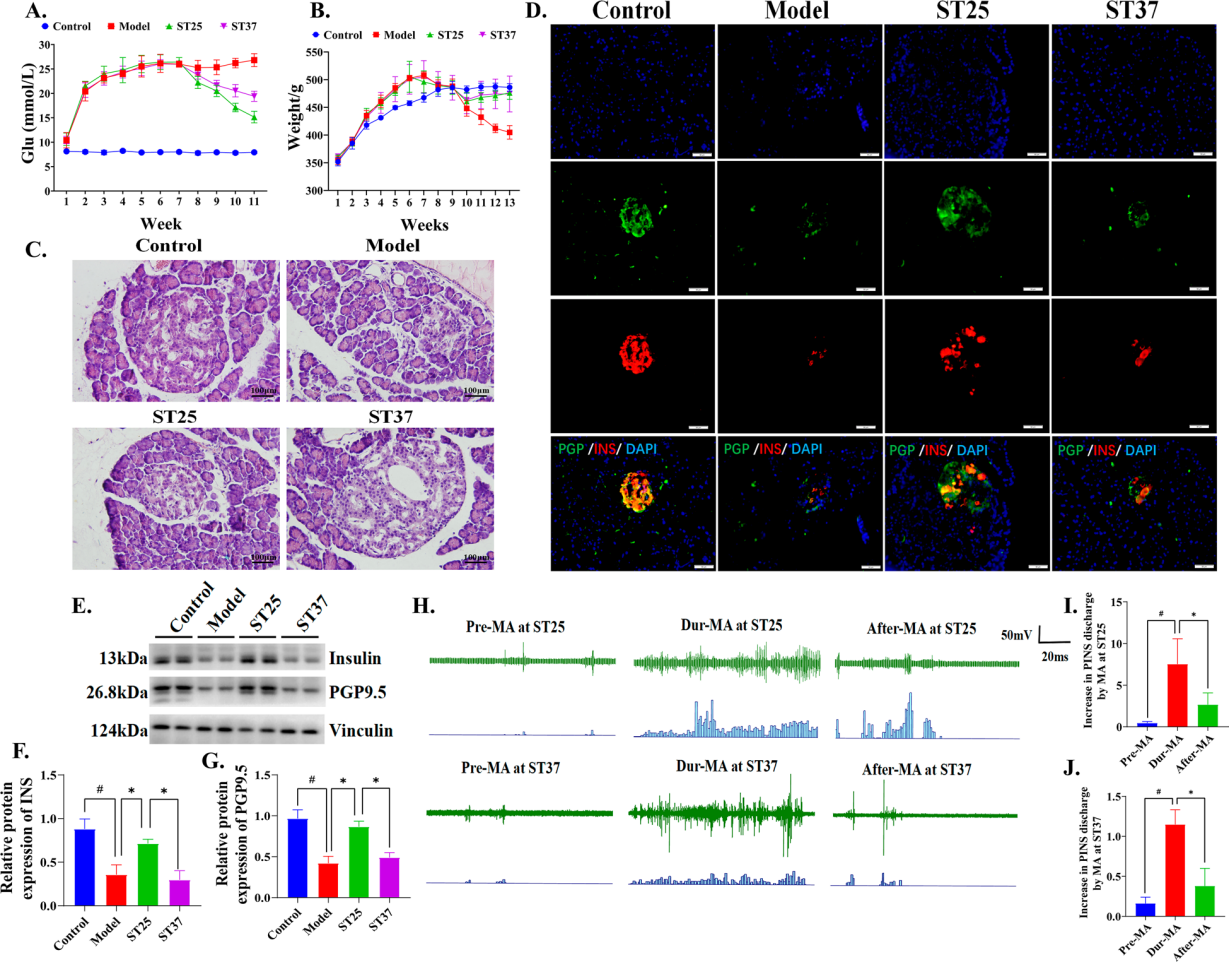
EA at ST25 regulates PINS to repair pancreatic function. **A** Random blood glucose levels of rats during experiment. **B** Weight of rats during experiment. **C** Representative HE images of the pancreas among groups. **D** Representative IF images of the pancreas among groups. DAPI stained the nuclei (blue), the green immunofluorescence represents the pan-neuronal marker protein gene product 9.5 (PGP9.5) and the red immunofluorescence represents the insulin. The orange color in the merged pictures indicates co-expression. Islets were observed under a microscope (× 400 magnification). Scale bar = 50 μm. Four groups share scale bars. **E**, **F** Effect of EA on insulin and **E**, **H** Pan-neuronal marker protein gene product 9.5 (PGP9.5) expression. Vinculin was used as an internal reference protein, ^#^*P* < 0.05 vs. normal control, ^*^P < 0.05 vs. model or another treatment group. **H**, **I** Change in PINS discharge frequency after MA at ST25 or **H**, **J** ST37. *PINS* pancreatic intrinsic nervous system, *ST25* Tianshu acupoint, *ST37* Shangjuxu acupoint, *MA* manual acupuncture, *INS* insulin, *PGP9.5* protein gene product 9.5

The hyperglycemia of the model group was maintained during the treatment, which lasted for 4 weeks. The blood glucose level in the EA at ST25 or ST37 group decreased from the 2 week of treatment (Fig. 2A). While the weight of both treatment groups increased since then (Fig. 2B). Moreover, these effects persisted for 3 weeks until the end of the experiment. The curative effect of EA at ST25 or ST37 as Fig. S1A-B showed that 4 weeks of EA at ST25 or ST37 improved glucose tolerance of HFD-STZ-induced T2DM rats. The fasting plasma glucose (FPG), insulin levels, HOME-IR, HbA1c and Leptin of the model group were significantly higher than that in the control rats (Fig. S1C-G). EA at ST25 or ST37 respectively leaded to a decrease in these indictors in comparison with the values in model group. Moreover, the FPG of ST25 group is lower than that of ST37 group. Notably, the HOMA-β, HOMA-IS and glucagon-like peptide-1 (GLP-1) of the model rats were much lower than in the control rats (Fig. S1H-J). However, EA at ST25 leaded to higher HOMA-β and HOMA-IS than the model group. While EA at ST37 did not have such effect. Meanwhile, EA at ST25 or ST37 differently repaired the systemic glycolipid metabolism level and promoted the pro-inflammatory/anti-inflammatory balance in the T2DM, and the ST25 effect was more favorable (Fig. S1K–O).

### EA at ST25 or ST37 restored islet morphology and function through PINS

HFD-STZ-induced T2DM rats showed moderate damage and swelling of pancreatic β cells as Fig. 2C. The islet shape was round and regular in the treatment groups. Further observation through immunofluorescence staining verified the pathological change in pancreatic β cells of model rats. Moreover, the pancreatic intrinsic nervous system (PINS) was damaged during HFD-STZ-induced T2DM as Fig. 2D. The expression of pan-neuronal marker protein gene product 9.5 (PGP9.5) in the pancreatic tissue of the model group was significantly lower than that in the control rats (Fig. 2E, G). EA at ST25 resulted in an increase in PGP9.5 expression in comparison with model group. While EA at ST37 did not have those effects. The co-expression of insulin and PGP9.5 revealed EA at ST25 or ST37 restored islet morphology through PINS.

### EA restored pancreatic endocrine function through recovery of sympathovagal balance via β_2_-AR

Sympathovagal balance is seen as an indicator of differences in autonomic nervous system response under T2DM [33]. Thus, monitoring and restoring this balance can help control disease progression, such as assessing changes in the transmitters released by various neuronal subtypes. The level of choline acetyltransferase (ChAT) expression decreased in the model group (Fig, 3A-B), while level of tyrosine hydroxylase (TH) increased in the model group (Fig, 3A, C), indicating pancreatic autonomic nerve remodeling. The expression of these neurotransmitters reversed after EA at ST25, however, EA at ST37 did not have such effect. The alternation of LF, HF and LF/HF ratio can help to determine the potential pathological changes of the system under the state of hyperglycemia. Frequency domain analysis of HRV revealed a sharply attenuation of LF norm and HF norm, on the contrary, augmented the levels of LF/HF ratio (Fig. S1P, Table S2). These results suggest typical changes dominated by sympathetic remodeling in T2DM. Further, acupuncture restraint of autonomic abnormal functions in HFD-STZ-induced diabetes rats. It is suggested that sympathetic nerve activity is involved in EA regulation of pancreatic function.

**Fig. 3:**
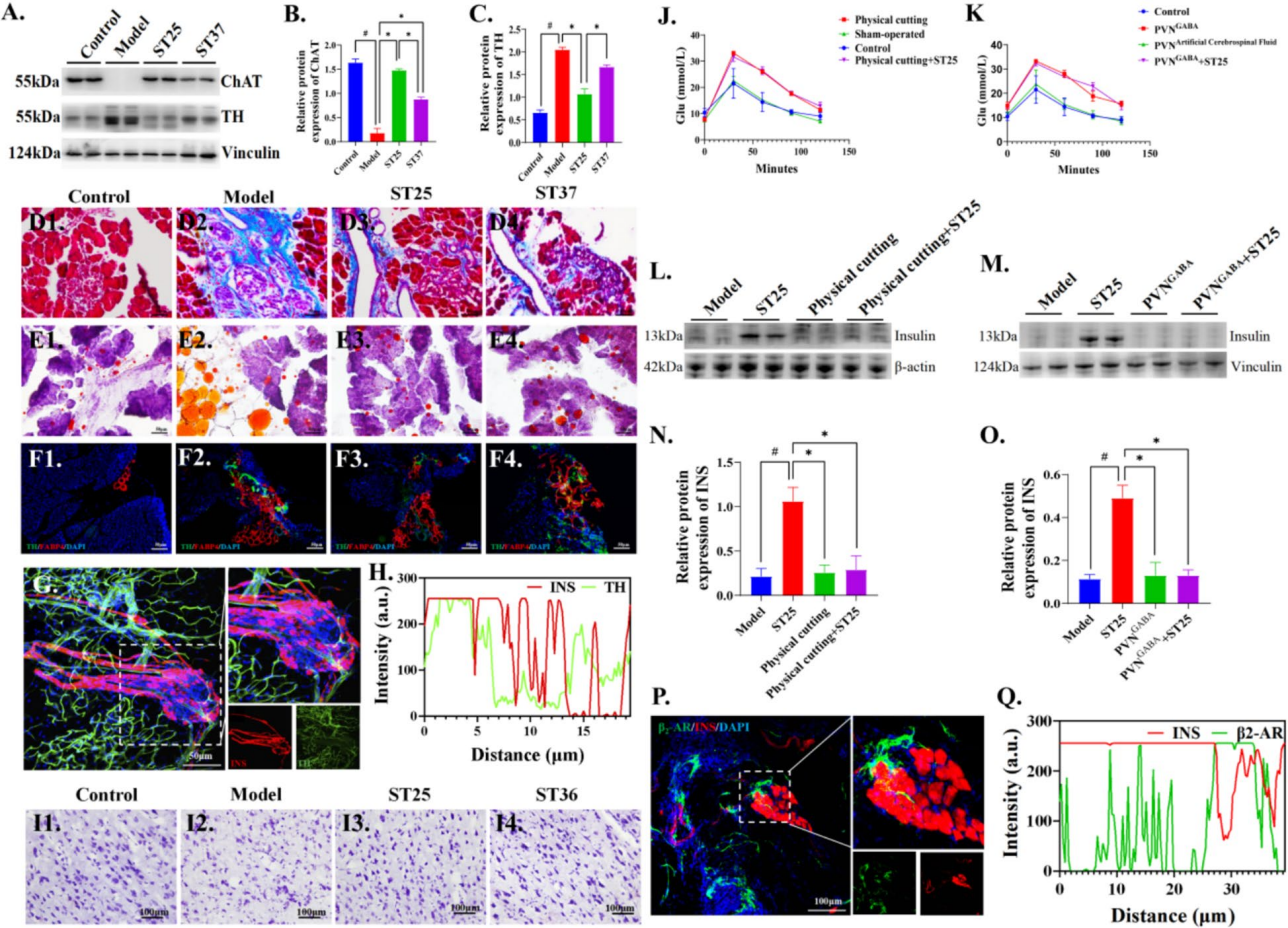
EA at ST25 repairs pancreatic function and is β_2_-AR-dependent. **A**, **B** Effect of EA on choline acetyltransferase (ChAT) and **A**, **C** Tyrosine hydroxylase protein (TH) expression. Vinculin was used as an internal reference protein. **D** Differences in pancreatic fibrous degeneration and **E** Steatosis in rats in each group. **F** Representative IF images of the pancreas among groups. DAPI stained the nuclei (blue), the green immunofluorescence represents the TH and the red immunofluorescence represents the FABP4. **G** Representative IF images and **H** Quantitative fluorescence analysis of the pancreas in model group. DAPI stained the nuclei (blue), the green immunofluorescence represents the CGRP and the red immunofluorescence represents the insulin. **I** Differences in PVN Nysted staining in rats of various groups. **J** Changes in glucose tolerance in rats after PVN injection of GABA and **K** Excision of pancreatic sympathetic nerve. **L**, **N** Differences in INS content expression in pancreatic tissues after PVN injection of GABA and **M**, **O** Excision of pancreatic sympathetic nerves. **P** Representative IF images and **Q** Quantitative fluorescence analysis of the pancreas in model group. DAPI stained the nuclei (blue), the green immunofluorescence represents the β2-AR and the red immunofluorescence represents the insulin. ^#^*P* < 0.05 vs. normal control, ^*^P < 0.05 vs. model or another treatment group. EA, electroacupuncture; ST25, Tianshu acupoint; PVN, hypothalamic paraventricular nucleus; GABA, gamma aminobutyric acid

Therefore, the pancreatic sympathetic nerve was used to record the changes of electrophysiological activity to clarify the relationship between manual acupuncture (MA) and PINS. MA at ST25 (7.52 ± 6.10 Hz) was significantly increased compared to the pre-MA frequency (0.46 ± 0.36 Hz, *P* < 0.05, Fig. 2H–I) in HFD-STZ-induced diabetes rats. The activity of PINS during MA at ST37 was enhanced PINS discharge frequency (1.15 ± 0.41 Hz), which was obviously different from that of pre-MA (0.17 ± 0.17 Hz, *P* < 0.05, Fig. 2H, J). MA at ST25 has the potential to regulate the sympathetic nervous activity of the pancreas. By pancreatic whole tissue immunofluorescence, it was observed that HFD-STZ-induced diabetes rats pancreatic steatosis was associated with changes in sympathetic nerve activity (Fig. 3D–F). This suggested a sympathetic-dependent of EA at ST25-mediated pancreatic lipid reprogramming.

Further, we used central chemical blockade and peripheral physical excision of sympathetic nerves to clarify the neuroanatomical pathways of EA in regulating pancreatic endocrine secretion. We found severe neuronal damage in the hypothalamic paraventricular nucleus (PVN) of HFD-STZ-induced diabetes rats (Fig, 3I). After PVN injection of gamma aminobutyric acid (GABA) to inhibit sympathetic efferents, glucose tolerance was markedly abnormal (Fig. 3K) and insulin secretion was counteracted by the regulation of insulin secretion by EA at ST25 (Fig. 3M, O), the carrier control group did not show this phenomenon (Fig. S1R). The sympathetic components of the plexus pancreaticus capitalis were further excised to clarify the effect. After resection of the pancreatic sympathetic nerves, it showed a similar phenomenon to PVN injection of GABA (Fig. 3J, L, N, S1Q). Further by pancreatic whole-tissue immunofluorescence, we observed that insulin secretion was closely associated with β_2_-AR expression, which revealed a β_2_-AR-dependent regulation of pancreatic function by EA at ST25 (Fig. 3P–Q).

### Pancreatic endocrine function was restored through the TRPV1-CGRP-β cell circuit

**Fig. 4:**
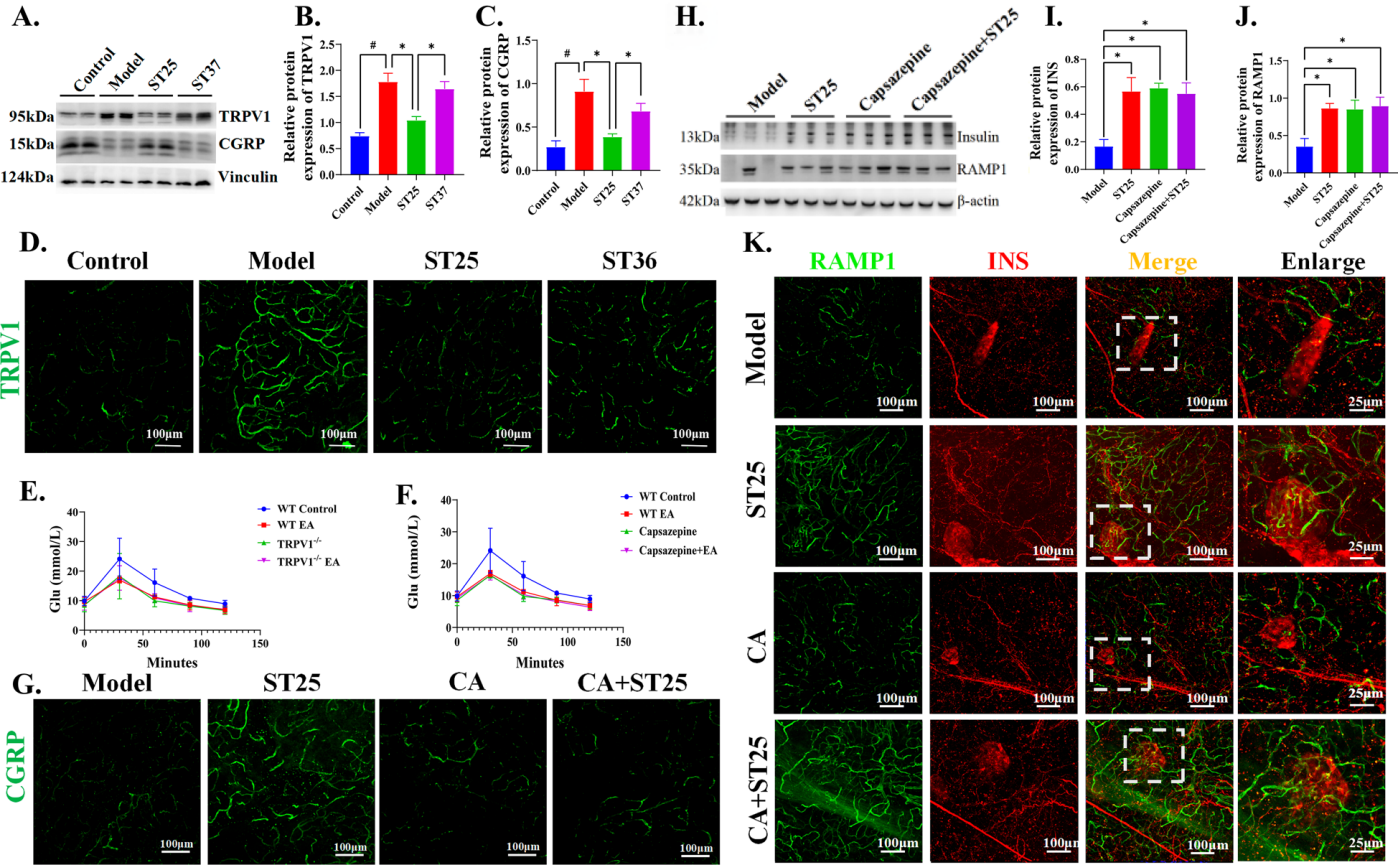
TRPV1-CGRP-β cell pathway mediates improvement of pancreatic glucose metabolism by EA at ST25. **A**, **B** Effect of EA on transient receptor potential vanilloid 1(TRPV1) and **A**, **C** Calcitonin gene–related peptide (CGRP) expression. Vinculin was used as an internal reference protein, **D** Representative IF images among groups. DAPI stained the nuclei (blue), the green immunofluorescence represents the TRPV1. **E** Changes in glucose tolerance in TRPV1-/- mice and **F** Pancreatic-biliary duct-injected capsazepine rats. **G** Representative IF images among groups. DAPI stained the nuclei (blue), the green immunofluorescence represents the CGRP. **H**, **I** Effect of EA on insulin and **H**, **J** receptor activity modifying protein 1 (RAMP1) expression. β-actin was used as an internal reference protein, **K** Representative IF images among groups. DAPI stained the nuclei (blue), the green immunofluorescence represents the RAMP1 and the red immunofluorescence represents the insulin. ^#^*P* < 0.05 vs. normal control, ^*^P < 0.05 vs. model or another treatment group

For further investigations, the expression of markers of sensory neurons has been observed. We focused on the expression of calcitonin gene–related peptide (CGRP) since it can be regulated through transient receptor potential vanilloid 1(TRPV1) [7]. In comparison with the normal control group, the model group showed an increased expression of TRPV1 and a reduced expression were found in the ST25 group (Fig. 4A, B, D). Conversely, the expression of calcitonin gene-related peptide (CGRP) was markedly increased following electroacupuncture (EA) at ST25 (Fig. 4A, C). We subsequently investigated the role of the TRPV1-CGRP pathway in mediating the glucose-lowering effects of EA at ST25 by systematically knocking down and chemically silencing local TRPV1 sensory afferent nerves in the pancreas. Our findings indicated that EA at ST25 did not exhibit hypoglycemic effects in TRPV1^−/−^ mice or in rats with chemically silenced pancreas (Fig. 4E–F), and the regulation of CGRP-positive nerves was not significant (Fig. 4G). This suggests that the TRPV1-CGRP pathway is a crucial molecular mediator for EA at ST25 in regulating pancreatic glucose-lipid metabolism. CGRP, by binding to its receptor, is involved in the regulation of insulin secretion. To further elucidate the specific mechanism by which the TRPV1-CGRP pathway enhances insulin secretion, we examined the CGRP receptor receptor activity modifying protein 1 (RAMP1) on pancreatic islets. We observed that RAMP1 expression was significantly diminished in the islets of model rats but was upregulated following EA at ST25. Notably, the EA-induced effect was abolished after local TRPV1 nerve silencing with capsazepine (Fig. 4H–K).

### EA at ST25 mediated glial cells pruning of pancreatic TRPV1 axons

**Fig. 5:**
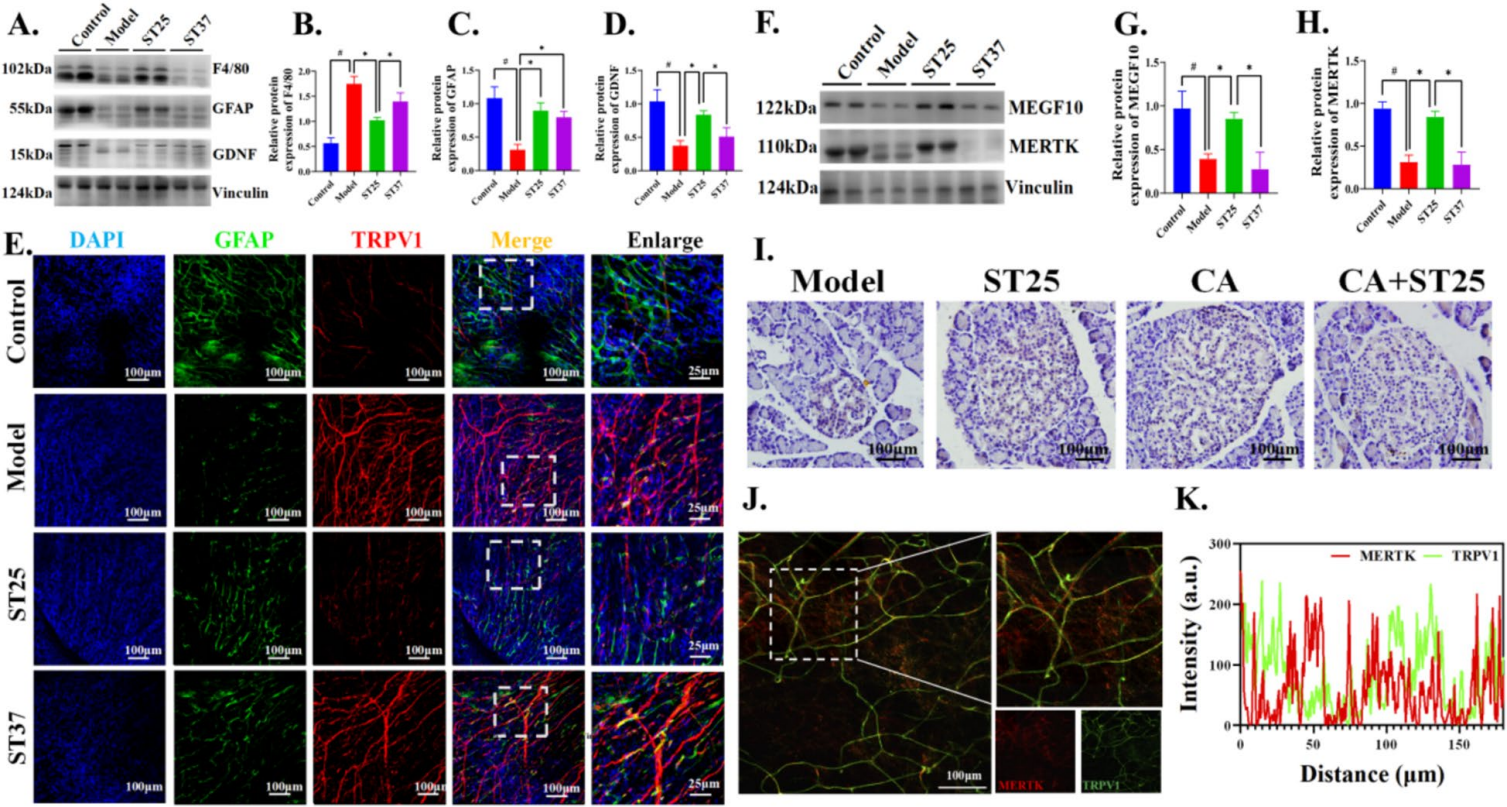
Glial cell-derived repair of TRPV1 neural axons to mediate EA to improve glycolipid metabolism. **A**, **B** Effect of EA on F4/80 (or EGF-like module-containing mucin-like hormone receptor-like 1), **A**, **C** Glial fibrillary acidic protein (GFAP) and **A**, **D** Glial cell-derived neurotrophic factor (GDNF) expression. Vinculin was used as an internal reference protein. **E** Representative IF images among groups. DAPI stained the nuclei (blue), the green immunofluorescence represents the GFAP and the red immunofluorescence represents the TRPV1. **F**, **G** Multiple EGF-like domains 10 (MEGF10) and **F**, **H** yeloid-epithelial-reproductive tyrosine kinase (MERTK) expression. Vinculin was used as an internal reference protein. **I** Typical IHC images of GDNF-positive labeling of the pancreas in various groups of rats. **J** Representative IF images and **K** quantitative fluorescence analysis of the pancreas in model group. The green immunofluorescence represents the TRPV1 and the red immunofluorescence represents the MERTK. ^#^*P* < 0.05 vs. normal control, ^*^P < 0.05 vs. model or another treatment group. *TRPV1* transient receptor potential vanilloid 1, *CA* capsazepine, *ST25* Tianshu acupoint

Previous experimental findings suggest that aberrant TRPV1 neuroactivity could result in a counterintuitive discordance between TRPV1 and CGRP expression patterns [34]. Glial cell-derived neurotrophic factor (GDNF) can be seen to increase to protect neurons [35]. We examined the restoration of TRPV1 nerves facilitated by glial cell sources to elucidate the dynamic interplay and correlation between TRPV1 and CGRP expression dynamics. The expression of F4/80 was decreased in the model group (Fig. 5A–B), and the expression of glial fibrillary acidic protein (GFAP) and GDNF were increased (Fig. 5A, C–D). After EA, the expression of these moleculars in ST25 was reversed, as shown in Fig. 5. ST37 only restored the expression of GDNF, but had no significant effect on the expression of F4/80 and GFAP. These indicating the cascade response of pancreas to the inflammatory state and the adaptive changes of peripheral glial cells. The regeneration of TRPV1^+^ nerve fibers by GFAP^+^ pancreatic glial cells was notably enhanced following EA at ST25, with minimal changes observed in the ST37 group (Fig. 4E). Astrocytes play a pivotal role in the process of synaptic pruning by expressing phagocytic receptors, including MEGF10 and MERTK. Our findings indicate that Electroacupuncture (EA) applied at the ST25 acupoint can significantly enhance the expression levels of both multiple EGF-like domains 10 (MEGF10) and myeloid-epithelial-reproductive tyrosine kinase (MERTK). This suggests that EA at ST25 may have a regulatory effect on astrocyte-mediated synaptic pruning, potentially contributing to the optimization and refinement of neural circuitry (Fig. 5F–H). The pancreatic MEFG10 modulation induced by EA at ST25 was significantly reduced upon local TRPV1^+^ nerve fiber inhibition using capsazepine (Fig. 5I). Meanwhile, we further found that MERTK acts in overactive TRPV1^+^ nerve axons in the HFD state (Fig. 5J–K). These findings imply a close interaction between glial cell sources and TRPV1^+^ nerve axons, which collaboratively contribute to the ameliorative impact of EA at ST25 on glucolipid metabolism.

### EA at ST37 mediates enteropancreatic interactions via GLP-1

**Fig. 6:**
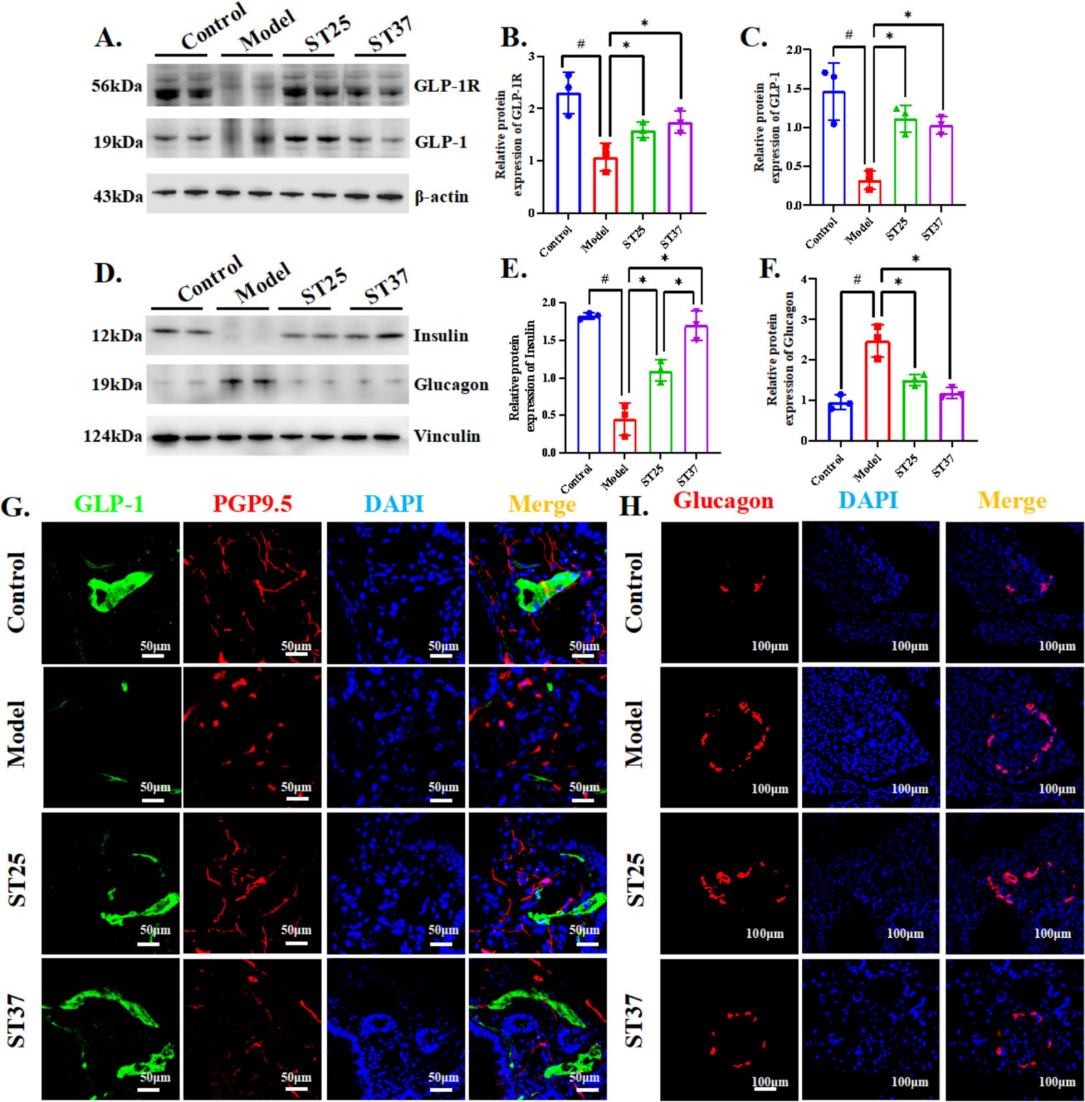
EA at ST37 mediates enteropancreatic interactions via GLP-1. **A**, **B** Effect of EA on GLP-1R and **A**, **C** GLP-1 expression. β-actin was used as an internal reference protein. **D**, **E** Effect of EA on insulin and **D**, **F** Glucagon expression in colon tissue. Vinculin was used as an internal reference protein in colon tissue. **G** Representative IF images of the pancreas among groups in colon tissue. DAPI stained the nuclei (blue), the green immunofluorescence represents the GLP-1 and the red immunofluorescence represents the PGP9.5. **H** Representative IF images of the pancreas among groups in colon tissue. DAPI stained the nuclei (blue), the red immunofluorescence represents thglucagon. Scale bar = 100 μm. Four groups share scale bars. ^#^*P* < 0.05 vs. normal control, ^*^P < 0.05 vs. model or another treatment group. GLP-1, glucagon-like peptide-1; GLP-1R, glucagon-like peptide-1 receptor; PGP9.5, protein gene product 9.5

Although the reparative impact on pancreatic inflammation for the ST37 group was less pronounced compared to the ST5 group, it nonetheless exerted a measurable effect on pancreatic function regulation, particularly in its influence on GLP-1 secretion (Fig. S1J). GLP-1, predominantly secreted by intestinal L cells, facilitates insulin release and curbs glucagon secretion in a glucose-dependent manner. This led us to further investigate whether EA at ST37 could enhance pancreatic function via the modulation of enteropancreatic reflexes. Our findings indicated that EA at ST37 notably increased the levels of GLP-1, the GLP-1 receptor (GLP-IR), insulin, and glucagon within colonic tissues, with the intestinal insulin regulatory effect outperforming that of the ST25 group (Fig. 6A–F). Immunofluorescence co-localization revealed a substantial rise in GLP-1 positive nerves in the colon following EA intervention at ST37 (Fig. 6G), which exhibited a dynamic correlation with the reduced glucagon levels in the pancreas (Fig. 6H).

## Discussion

### EA can repair sympathovagal balance-associated β cells dysfunction

Although the HFD-STZ-induced T2DM model rats effectively replicate key pathological features of insulin resistance and progressive β-cell destruction observed in human T2DM, they fail to fully recapitulate the complexity of human T2DM. This includes differences in genetic background, immune response, and the slower progression of human T2DM, which is often accompanied by more complex associated complications compared to those in HFD-STZ-induced T2DM model rats [36]. Therefore, the present study focuses on adaptive changes in in the pancreatic nervous system observed in both human and rodent pancreatic samples of T2DM [37], aiming to minimize discrepancies caused by interspecies differences and modeling factors. Commonly, in the pancreatic islets, the parasympathetic neurons promote insulin secretion while the sympathetic neurons inhibit insulin secretion [38, 39]. We observed a marked increase in parasympathetic activity and a decrease in sympathetic activity. Moullé [40] discovered that parasympathetic neurons stimulate β cells proliferation, while sympathetic signals inhibite β cells proliferation. This suggested that autonomic nervous system may mediate the association between β cells and glucose metabolism. Our results showed that the ratio of TH and ChAT was imbalanced in hyperglycemia, indicating that both the sympathetic and vagus nerves were impaired during T2DM. EA corrected the abnormal expression of TH and ChAT. Acupuncture, as one of the appropriate stimulations to regulate the autonomic nervous system [41], can correct abnormal nerve abnormal and restore sympathovagal imbalance. The HRV parameter changes were consistent with the phenomenon of pathological changes in both TH and ChAT neurotransmitters in Western Blot (Fig. 3, Fig. S1). After treatment, HFD-STZ-induced diabetic neuropathy significantly improved, implying stimulation at ST25 or ST37 plays a positive role, and the effect of ST25 is better than that of ST37 (Table S1). The low LF/HF ratio could be observed in HFD-STZ-induced diabetes, which focused on the remodeling of the sympathetic nerve [42]. Hyperglycemia may lead to cardiac and systemic autonomic dysfunction, since the growth and repair of nerve fibers will be affected by endocrine cells [43]. Moreover, the effect pathway was realized through increased oxidative stress [44] and local accumulation of inflammatory factors, indicate a two-way relationship between hyperglycemia and nerve damage. Thus, our results demonstrated that autonomic nerve regulation injury can contribute to the development of diabetes, and this damage can be partially reversed by acupuncture. Perciaccante [45] proposed that there was a linear correlation between sympathetic overactivity and increased insulin resistance. It indicates that early treatment of insulin resistance is associated with autonomic dysfunction and recovery of neurological function, especially the sympathetic nervous. Based on the mentioned theories [42, 45], we speculate that EA at ST25 had better effects than ST37 in correcting pancreas nerve abnormalities and improving endocrine.

The pancreatic nervous system is remodeled in response to stress-driven by high glucose metabolism and local inflammation, then reconstructs regulatory circuits with β cells. The neuro-correlational changes observed in the pancreas were consistent with that of the HRV, showing typical redistribution of TH. These results keep with the perception that parasympathetic neurons directly influence β cell function.

### EA reverses β cell function through “TRPV1 receptor depletion” hypothesis

**Fig. 7:**
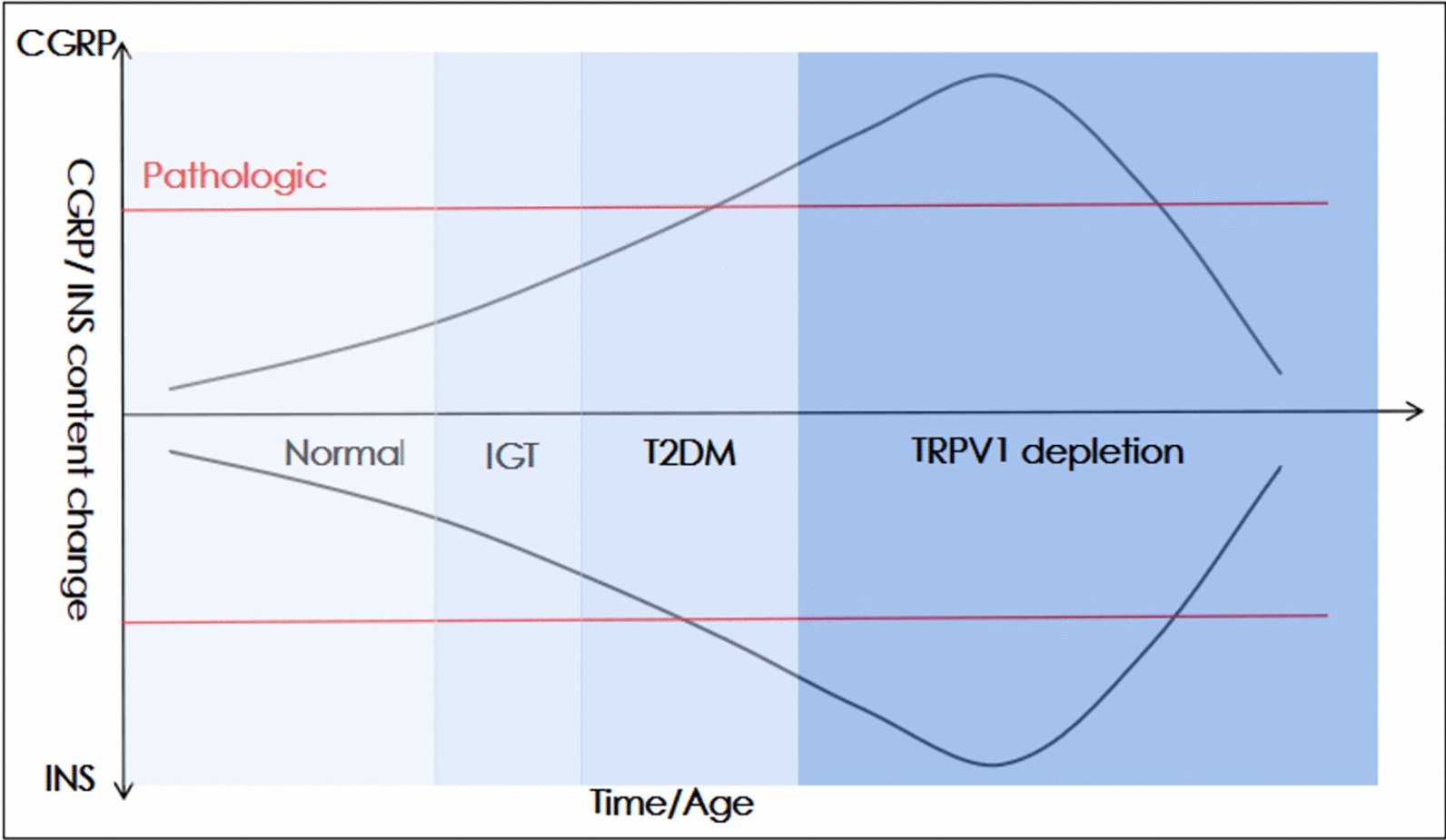
TRPV1-CGRP-β cell circuit damage leads to T2DM and the “Receptor Depletion” hypothesis. The release of CGRP (located in TRPV1 positive nerve endings) and the secretion of insulin may be involved in the regulation of glucose homeostasis. During T2DM, continuous low-grade tissue inflammation increases the expression of CGRP and inhibits insulin secretion. However, the secretion of insulin from β cells remains at an extremely high level due to peripheral insulin resistance. Prolonged stimulation of the TRPV1 receptor shows a severe decrease in activity until exhaustion, which visually manifests a sharp attenuated in CGRP secretion. Correspondingly, the insulin secretin is at low level. CGRP, calcitonin gene-related peptide; INS, insulin; IGT, impaired glucose tolerance. (Figure adapted from D.X. Gram, 2017 [7].)

**Fig. 8:**
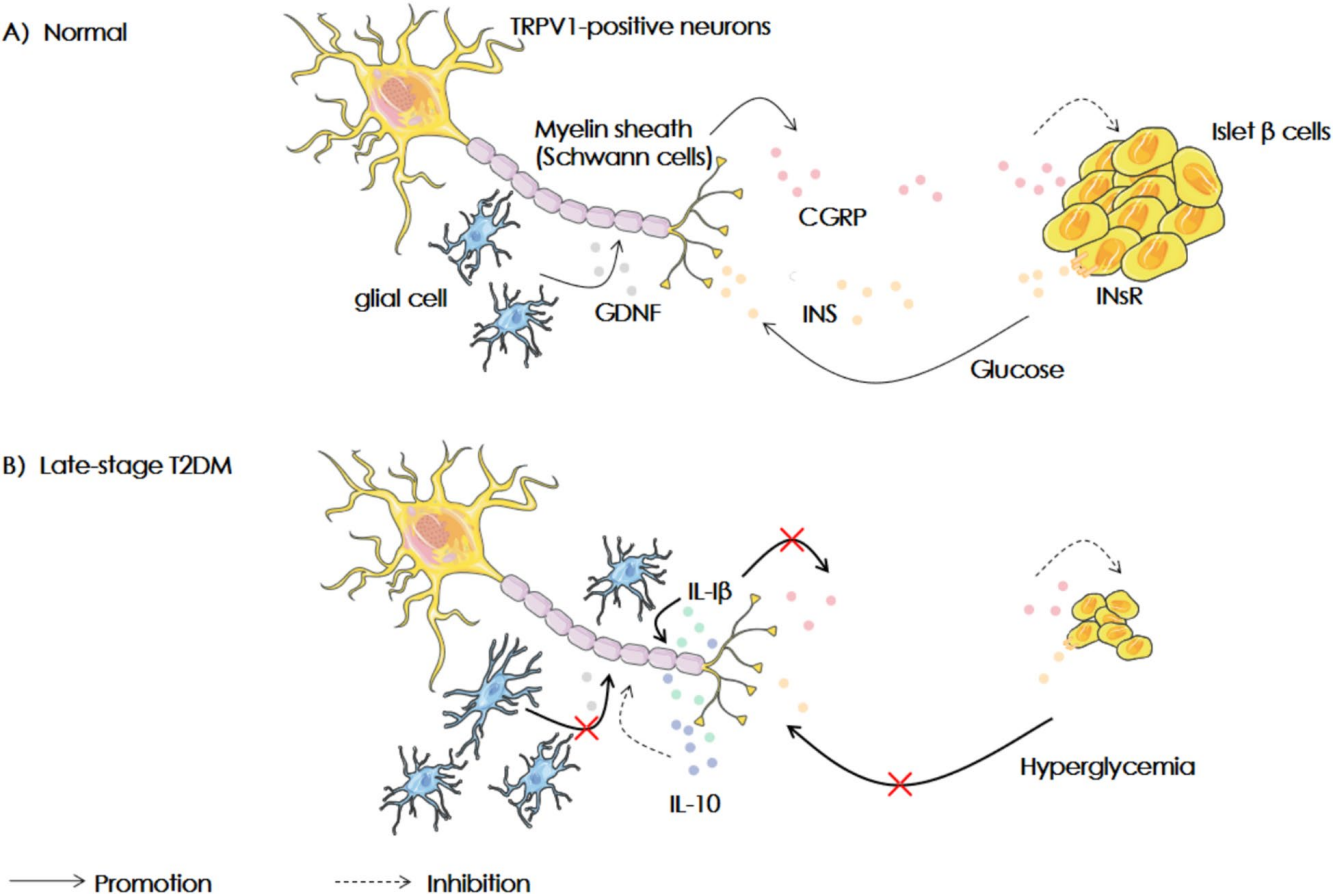
The “Receptor Depletion” hypothesis: The evolution of the transient receptor potential vanillin-1 (TRPV1) receptors in Type 2 Diabetes (T2DM). The transient receptor potential vanillin-1 (TRPV1) positive neurons containing CGRP can control the pancreatic β cells. They form a feedback loop formed which is affected by glial cells and inflammatory cytokines. After continuous chronic stimulation, β cell function was seriously impaired and insulin secretion decreased. At this time, TRPV1 receptor will be inactivated, affecting CGRP secretion and aggravating T2DM. INS, insulin; INsR, insulin receptor; CGRP, calcitonin gene-related peptide; GDNF, glial cell-derived neurotrophic factor; IL-1β, interleukin-1β; IL-10, interleukin-10

The adaptive proliferation of TRPV1 was more obvious in the pancreatic nervous system. The endings of TRPV1-positive neurons can release CGRP in the rat pancreas [46]. The function of TRPV1 is linearly correlated with insulin sensitivity [47]. However, the changes in TRPV1 receptor activity may affect the release of CGRP. Advanced diabetic neuropathy may also confirm these findings [48]. TRPV1 receptors were also significantly damaged when insulin sensitivity was severely impaired [49]. Moreover, the co-localization of insulin receptor (InsR), TRPV1, and CGRP suggested that insulin regulates the growth or function of the sensory nerve through specific neuropeptides [50]. We also noticed that the trend of InsR impairment in the pancreas was consistent with that of TRPV1, which in keep with the above conclusion. These results suggest that TRPV1 may be the local control center for the rapid regulation of hormone secretion, especially for insulin and other endocrine hormones, and form a two-way interaction. Interestingly, we found that in HFD-STZ-induced T2DM rats, the expression of TRPV1 was significantly enhanced, but CGRP was insufficient. Our results were consistent with Gram’s study [7]. According to Tang’s claims, the activity of TRPV1/CGRP in T2DM is nonlinear (Fig. 7). Combined with the above findings, we propose that although the protein expression level of TRPV1 was sharply augmented during T2DM, the activity of TRPV1 might remain unchanged or even obviously attenuated, suggesting the presence of masses of passivated receptors [7]. At this time, neuropathy and impaired insulin secretion may still be evident, we can observe severe T2DM manifestations likewise [6]. Under normal conditions, TRPV1 positive neurons release CGRP to directly inhibit insulin release. It forms a local feedback mechanism with β cells to ensure that insulin release and synthesis are in balance. Meanwhile, glial cells release GDNF to maintain the balance of the TRPV1 receptor (Fig. 8) [51]. Hyperglycemia state can reduce the release of GDNF, thereby mitigating the protective effect of transmitters and causing neurotoxicity [52]. In T2DM, the TRPV1 receptor is overactivated and its activity decreases sharply. This will lead to a pathological condition that does not match the secretion of CGRP. At this time, the GDNF secreted by glial cells becomes more adaptive to repair the TRPV1 receptor [53]. The anti-inflammatory/pro-inflammatory balance of IL-10/IL-1β also helped to restore TRPV1 activity (Fig. 8A). However, those pathways have physiological limits that excessive stimulation may deplete/aggravate the pathological manifestations (Fig. 8B) [53]. While TRPV1 receptor passivation ultimately determines that the above stimulation has little effect, even if the increase of TRPV1 receptor cannot promote the secretion of downstream neurotransmitters, and eventually abortive.

### Glial Cells: EA-mediated Effects of Peri-islet Components on TRPV1-CGRP-β cell Circuits

In sophisticated neural networks, synaptic connectivity and activity are meticulously balanced, maintaining a state of dynamic equilibrium that is contingent upon the relentless pruning and clearance of inactive synapses by glial cells [54]. This ongoing process of synaptic pruning, which extends throughout an organism's lifespan, is particularly evident in the cortex, hippocampus, and neuromuscular junctions of the peripheral nervous system [55]. Such pruning is crucial for the maturation and remodeling of synapses, the functionality of neurons, and the establishment and preservation of neural circuitry. Astrocytes, a type of glial cell, can facilitate synaptic pruning through the action of two phagocytic receptors: MEGF10 and MERTK. Both MEGF10 and MERTK are instrumental in mediating this pruning process. Mice deficient in Megf10 and Mertk exhibit synaptic overabundance reminiscent of those lacking the complement component C3, indicating a critical role for these receptors in maintaining synaptic balance [43, 56]. The pruning mediated by MEGF10 and MERTK in astrocytes specifically targets and eliminates weakly active synapses, thereby ensuring the fidelity and adaptability of neural circuits. Furthermore, MEGF10 has been identified as a receptor for C1q, playing a pivotal role in astrocytes' phagocytic clearance of apoptotic cell debris. In the absence of Megf10, as seen in knockout mice, there is an increase in apoptotic neurons and a concurrent decrease in astrocyte-mediated phagocytosis [57]. This highlights the multifaceted role of MEGF10 in maintaining neural health and underscores the importance of glial cells in the intricate dance of synaptic life and death. Our results indicated that EA at ST25 or ST37 increased the level of GDNF, respectively. EA ameliorated the release of CGRP and enhanced TRPV1 expression. EA at ST25 showed therapeutic benefits. Anand [58] proposed that GDNF supplementation can enhance TRPV1 activity. The correlation between GDNF deprivation and TRPV1-positive axonal degeneration was highlighted, which is consistent with our observation. Inflammatory factors such as TNF-α can stimulate glial cells to release GDNF [59]. However, the metabolic stress caused by HFD induced glial inactivation and aggravated islet inflammation. It was in keeping with the attenuated trend of GFAP which is expressed in rat islets. Consistent with this, severe impairment of GNDF and β cell function were observed in the model group. Ultimately, EA exerts protective effect towards the β cell by restoring the local feedback in TRPV1-CGRP-β cells, which is involved in GDNF mediation. Nonetheless, the related pathways of EA-mediated GNDF to repair TRPV1 function remain to be studied. The secretion mismatch of TRPV1-CGRP was improved partially after EA at ST25 rather than ST37. Since TRPV1 is the transmitter of upstream signals, we focused on the repair of TRPV1 activity, thus the therapeutic effect of EA at ST25 was more significant. The mechanism of EA at ST25 may be jointly affected by the aforementioned sympathetic pathways and inflammatory factors (Fig. 8).

### GLP-1 pathway: a feasible way for the treatment of T2DM by EA at ST37

Stimulation of the ST37-mediated entero-pancreatic crosstalk may represent a novel therapeutic strategy for modulating pancreatic function. One of the peripheral targets of insulin is the intestinal tract, which regulates its sensitivity and responds appropriately [60]. Meanwhile, the intestinal tract can secrete GLP-1 to regulate the release of pancreatic β cells [61]. Intestinal type 2 innate lymphoid cells can migrate to the pancreas, where they respond to neural signaling to enhance pancreatic endocrine function and improve glycemic control[62]. These studies suggested that both the intestine and pancreas are critical organs for the maintenance of metabolic homeostasis, and that there is a dynamic functional exchange between them. GLP-1 is released after nutrient intake and acts in conjunction with hyperglycemia in a synergistic way to stimulate insulin secretion. β cell function is deficient in diabetic rats, and GLP-1 release is attenuated or even disappeared [63]. Our study replicated the results of low serum GLP-1 levels in diabetic rats (Fig. S1J). This observation indicates the synchronization of basal secretion between the intestine and the pancreas [64]. While the above changes can be restored after EA, specifically after EA at ST37. Therefore, EA-mediated entero-pancreatic GLP-1 secretion may be an underlying therapeutic mechanism of T2DM. Simultaneously, we noticed that EA at ST37 improved serum lipids and decrease inflammation. While dyslipidemia is the main driver of T2DM [65]. Serum leptin level was significantly correlated with percentage body fat [66] and PFG [67]. The metabolic effect of leptin antagonizes insulin [68]. These results suggest that leptin and its matched obesity status can be independent risk factors affecting T2DM, and the lipid-lowering effect of EA at ST37 can effectively treat T2DM. Considering the use of acupoints combination therapy, we advocate ST25 combined with ST37 stimulation. It exerts a steady anti-inflammatory and lipid-lowering effect, and might be related to the mechanism of entero-pancreatic interaction. For instance, the secretion of intestinal secretory cells can affect insulin release, alter adipose distribution, or overaccumulation [64].

### Limitations of the Study

There are several limitations in this research. The ambiguous relationship between the pancreatic innervation system limits our further discussion of sensory neurons [69]. There are endogenous and exogenous TRPV1 positive nerves in the pancreas of rats, and there are some potential differences in their functions [70, 71]. Thus, under the influence of the anatomical differences described above, CGRP content in the pancreas is mainly regulated by two factors: (a) External sources, dorsal root ganglion (DRG); (b) Internal origin, intra-pancreatic ganglion [72]. Therefore, we are not sure whether the nerve damage is the essential cause of the low levels in the pancreas. Future studies should complement the observation of local changes in pancreatic autonomic nerves and try to refine the subset of neurons activated by acupuncture and their interaction with glycometabolism.

## Supplementary Information

Supplementary material 1.

## Data Availability

The datasets used or analyzed during the current study are available from the corresponding author on reasonable request.
