# Supplementary material for: Electroacupuncture at ST25 mediated glial cells pruning of pancreatic TRPV1 neural synapse responds to neuropathy-associated beta cell dysfunction
Source: Chin Med. 2025 May 16;20:65. doi: 10.1186/s13020-025-01099-w (PMC12083143; doi:10.1186/s13020-025-01099-w)

**Table 1** Information on the use of primary antibodies

| **Antibody** | **Species** | **Dilution** | **Source** |
| --- | --- | --- | --- |
| Transient receptor potential vanilloid 1 (TRPV1) | Mouse | 1:1000 | Abcam |
| Choline acetyl transferase (ChAT) | Rabbit | 1:1000 | Abcam |
| PGP9.5 | Rabbit | 1:1000 | Abcam |
| Calcitonin gene–related peptide (CGRP) | Rabbit | 1:1000 | Abcam |
| Tyrosine hydroxylase (TH) | Mouse | 1:1000 | Santa Cruz |
| Glial cell-derived neurotrophic factor (GDNF) | Mouse | 1：1000 | Santa Cruz |
| Glial fibrillary acidic protein (GFAP) | Mouse | 1：1000 | Signalway Antibody |
| F4/80 | Mouse | 1：500 | Santa Cruz |
| Receptor activity modifying protein1 (RAMP1) | Mouse | 1：500 | Santa Cruz |
| Vinculin | Rabbit | 1:2000 | Abcam |
| β-actin | Rabbit | 1:2000 | Abcam |

**Table 2** Frequency-domain parameters of difference between normal rats and T2DM rats

|  | Normal | T2DM |
| --- | --- | --- |
| n | 6 | 7 |
| LFnorm, nU | 36.76±4.53 | 60.40±4.75^*^ |
| HFnorm, nU | 51.22±4.23 | 70.60±2.85^*^ |
| LF/HF | 0.71±0.18 | 0.29±0.04^*^ |

* P < 0.05, vs. normal group.

**Figure S1 PINS mediates EA to improve T2DM glucose-lipid metabolism.** (A) Differences in glucose tolerance and area under the (B) curve among groups of rats after intraperitoneal injection of 2 mg/kg glucose solution. (C) Changes in serum FPG, (D) insulin, (E) HOMA-IR, (F) HbA1c, (G) leptin, (H) HOMA-β, (I) HOMA-IS, (J) GLP-1, (K) IL-1β, (L) TNF-α, (M) IL-10, (N) TC, and (O) TG levels in rats in each group. (P) Effect of MA at ST25 or ST37 on HRV in normal or T2DM rats. (Q) Changes in glucose tolerance in rats after pancreatic sympathectomy and (R) PVN injection of GABA. ST25, Tianshu acupoint; ST37, Shangjuxu acupoint; MA, manual acupuncture; INS, insulin; LEP, leptin; PVN, hypothalamic paraventricular nucleus; GABA, gamma aminobutyric acid


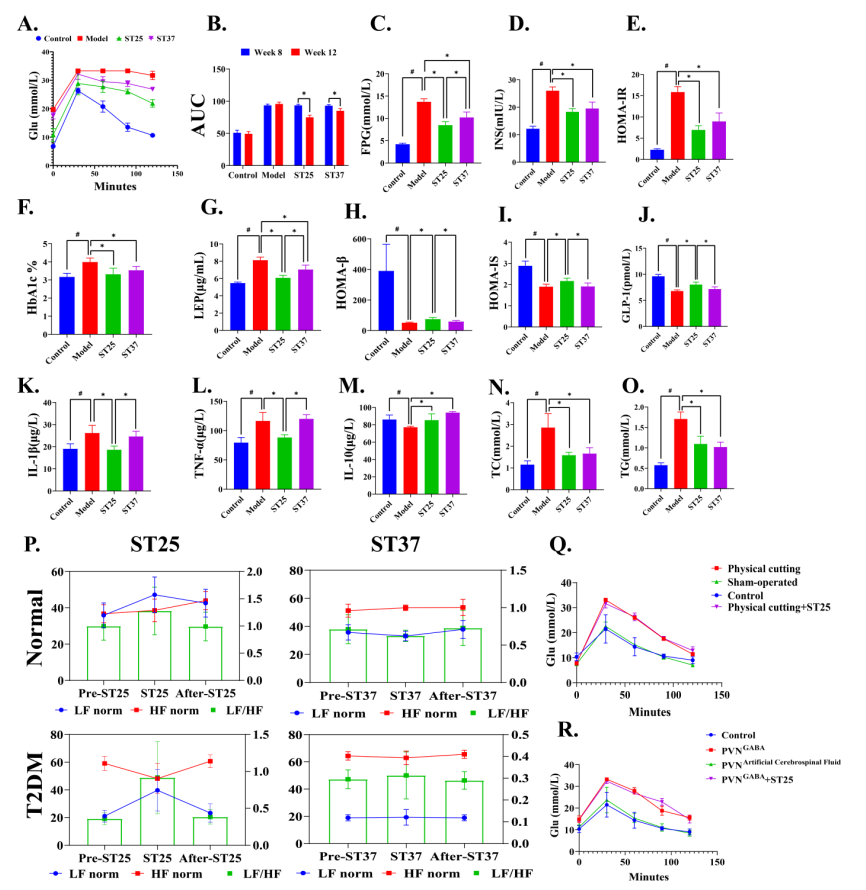

Supplement: Supplementary file 1 — Supplementary material 1. [file 13020_2025_1099_MOESM1_ESM.docx]
